# 

**Published:** 1893-07-01

**Authors:** 


### CONTENTS.

The Royal Wedding . . . . . . . . . . 209
Around the Hospitals . . . . . . . . . 210
Medical History from the Earliest Times. By E. T. Withington, M.A., M.B.Oxon. LX.—The Vitalists . . . 211
Contemporary Theory and Research . . . . . . 212
Diet in Disease. By Mrs. Ernest Hart. XXV.—On Thinning and Fattening . . . . . . . . . . 213
Gleanings in Science and Medicine . . . . . . 214
The Hospital Clinic—
Some Simple Uses of Carbolic Acid. I. . . . . . . 217
The Treatment of Fractures of the Nose . . . . 218
The Naphthols as Intestinal Anti-Infectants . . . 219
New Appliances and Things Medical.—Messrs. Allen and Hanbury's Preparations . . . . . . . . . 220
Annotations.—Neolithic Surgery—"I Decline to Meet You"—Hip Disease in Children . . . . . . . . . 215
Notes and News . . . . . . . . . . 216
The Institutional Workshop—
Within the Hospitals.—The Newcastle Eye Infirmary: By Our Special Commissioner . . . . . . . 221
Practical Departments.—Furniture for Nurses' Rooms . . 221
Festival Dinners and Meetings . . . . . . . . 222
Construction Notes . . . . . . . . . 223
Editor's Letter Box.—The Treatment of Scarlet Fever by Antiseptic Inunction . . . . . . . . . 224
Scraps and Gleanings . . . . . . . . . 224
The Book World of Medicine and Science . . . . vii
Medical Vacancies and Appointments . . . . ix

### EXTRA SUPPLEMENT.—THE NURSING MIRROR.

News from the Nursing World.—The Royal Wedding—Loyalty still Lives—Where are the Matrons?—Angels very much Disguised—A Permanent Home—Wise Local Government—Plain and Unattractive Duties—Scotch Branches of the Institute—Scotch Workhouse Nurses—New Nursing Arrangements in India—Opposed Progress—Chicago . . cxxxi

Sanitation for Nurses. By A Sanitary Inspector. XI.—Sanitary Provisions in Foreign Countries . . . . cxxxiii

Nursing in South Africa.—By Our Special Correspondent—Somerset Hospital, Cape Town . . . . . . cxxxiv

The Nurse Dolls—The Charter . . . . . . . cxxxv

The Royal British Nurses' Association . . . . cxxxv

Appointments . . . . . . . . . . cxxxv

India.—Mahableshwar—Drawbacks to a Hill Station . . cxxxvi

More Girl Nurses . . . . . . . . . . cxxxvi

Everybody's Opinion.—A Holiday Home Wanted—A Useful Suggestion . . . . . . . . . . . cxxxvi

Lady Roberts' Nursing Fund . . . . . . cxxxvi

Trained Nurses in Workhouses . . . . . . cxxxvi

Where to Go . . . . . . . . . . . cxxxvi

Precautions against Cholera.—Notes by a Nurse . . cxxxvii

For Reading to the Sick.—Riches . . . . . . cxxxvii

The Muse's Looking Glass.—The Reputation of George Saxon . . . . . . . . . . . . cxxxviii

Wants and Workers . . . . . . . . . cxxxviii

The Book World for Women and Nurses . . . . cxxxix

